# ^212^Pb: Production Approaches and Targeted Therapy Applications

**DOI:** 10.3390/pharmaceutics14010189

**Published:** 2022-01-13

**Authors:** Konstantin V. Kokov, Bayirta V. Egorova, Marina N. German, Ilya D. Klabukov, Michael E. Krasheninnikov, Antonius A. Larkin-Kondrov, Kseniya A. Makoveeva, Michael V. Ovchinnikov, Maria V. Sidorova, Dmitry Y. Chuvilin

**Affiliations:** 1 Physical and Chemical Technology Center, National Research Center Kurchatov Institute, 123182 Moscow, Russia; kokov_kv@nrcki.ru (K.V.K.); german_mn@nrcki.ru (M.N.G.); larkin_aa@nrcki.ru (A.A.L.-K.); makoveeva_ka@nrcki.ru (K.A.M.); chuvilin_dy@nrcki.ru (D.Y.C.); 2 Department of Chemistry, Lomonosov Moscow State University, 119991 Moscow, Russia; 3 Department of Regenerative Medicine, National Medical Research Radiological Center, 249036 Obninsk, Russia; ilya.klabukov@gmail.com; 4 Research and Educational Resource Center for Cellular Technologies, Peoples’ Friendship University of Russia, 117198 Moscow, Russia; krashen@rambler.ru; 5 Laboratory of Peptide Synthesis, National Medical Research Center of Cardiology, 121552 Moscow, Russia; peptide-mv@mail.ru (M.V.O.); MVSidorova@cardio.ru (M.V.S.)

**Keywords:** lead-212, thorium-228, radionuclide generator, α-radiation sources, targeted alpha therapy

## Abstract

Over the last decade, targeted alpha therapy has demonstrated its high effectiveness in treating various oncological diseases. Lead-212, with a convenient half-life of 10.64 h, and daughter alpha-emitter short-lived ^212^Bi (*T_1/2_* = 1 h), provides the possibility for the synthesis and purification of complex radiopharmaceuticals with minimum loss of radioactivity during preparation. As a benefit for clinical implementation, it can be milked from a radionuclide generator in different ways. The main approaches applied for these purposes are considered and described in this review, including chromatographic, solution, and other techniques to isolate ^212^Pb from its parent radionuclide. Furthermore, molecules used for lead’s binding and radiochemical features of preparation and stability of compounds labeled with ^212^Pb are discussed. The results of preclinical studies with an estimation of therapeutic and tolerant doses as well as recently initiated clinical trials of targeted radiopharmaceuticals are presented.

## 1. Introduction

The effect of targeted therapy on tumoral tissues is the crucial issue of modern fundamental and practical oncology. Targeted therapy is, essentially, the delivery of toxic preparations to cancer cells using artificially made biochemical bioconjugates that are able to selectively attach to the surface receptors of a cancer cell without affecting healthy tissues. Monoclonal antibodies and synthetic peptides, specific for various types of receptors expressed on the cancer cell surface, are used as targeting agents in radionuclide therapy [1]. Radionuclides such as ^177^Lu (*T_1/2_* = 6.7 d), ^90^Y (*T_1/2_* = 64 h), and a few other β-emitters are most often used for therapy and diagnostics [2].

Along with β-emitters in radionuclide therapy, α-emitting radionuclides are also used for cancer treatment. These have several advantages; namely, an α-particle has a shorter path length in tissue and high linear energy transfer, which most likely causes double-strand DNA, the simultaneous break of two complementary DNA strands in the same place, which causes the most toxic damage to a cell. Studies show that only a few passages of α-particles through a tumor cell are required to destroy it, while the surrounding healthy tissues are exposed to a low level of irradiation; therefore, α-emitters are especially effective in hematological diseases, micrometastases, and with areas of the cavity surfaces [3]. The following radionuclides can be used as α-emitters in targeted therapy: ^149^Tb (*T_1/2_* = 4.12 h), ^212^Bi (*T_1/2_* = 60 min), ^213^Bi (*T_1/2_* = 46 min), ^225^Ac (*T_1/2_* = 10 days), and some others. α-particles feature high energy (5–8 MeV) and a short path in substances (10–100 µm); therefore, localization of a sufficient amount of α-emitter ions near a tumor cell results in the selective destruction of malignant neoplasms with minimal damage to surrounding tissues. The development of radionuclide therapy targeted preparations labeled with different α-emitters has been in progress since the end of the 1980s and continues to the present with still greater dynamics [4].

**Figure 1:**
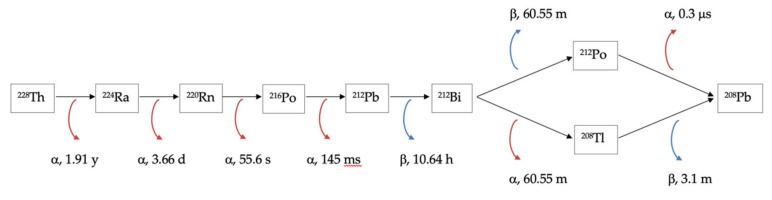
^228^Th decay chain scheme.

The application of short-lived radionuclides for the production of therapeutic complexes has certain unique features. For radionuclides to be effectively used over time, commensurable with their half-life period, it is necessary to produce radionuclides, perform complex formations with a bioconjugate, execute control of key parameters, and collect a preparation in the quantities required for introduction to the patient. This problem is solvable in two ways:

- Implementation of an automated module for radiopharmaceutical synthesis to perform all the operations necessary to provide the medical personnel with the synthesized preparation directly in a medical institution with a minimum expenditure of time;
- In vivo generators, which assume application of such radionuclides during labeling of bioconjugates, would generate the required daughter nuclide after introduction into the patient. The radionuclide of this kind for therapeutic ^212^Bi is ^212^Pb (*T_1/2_* = 10.64 h) β-emitter. ^212^Pb is of definite interest because its daughter nuclides (^212^Bi and ^212^Po) undergo α-decay, which allows us to view ^212^Pb as an in vivo generator of α-particles emitters. The radionuclide ^212^Pb belongs to a radioactive series of long-lived parent ^228^Th (*T_1/2_* = 1.9 year); see Figure 1.

The specific properties of certain radionuclide decay chains may play a key role in the production principle for its desired daughter radionuclides. For example, since ^228^Th contains gaseous ^220^Rn in its decay chain, its daughter ^212^Pb can be produced via ^220^Rn isolation. The ^224^Ra half-life period is rather convenient for ^224^Ra/^212^Pb generator implementation. For this reason, we tried to find ^212^Pb production techniques with scientific and industrial interest, namely, for radiopharmaceutical synthesis, investigations, and applications within nuclear medicine. One of the most important parameters in radiopharmaceuticals production is radionuclide purity because the injection of pharmaceuticals contaminated with long-lived parents into a patient could result in a fatal dose of radiation. Thus, we paid attention to this issue during the radionuclide-obtaining techniques review.

For a radiopharmaceutical to be used successfully, it must manifest sufficient stability to retain its targeting properties. Complex instability may result not only in a degradation of the targeting functions but also in the formation of radioactive compounds capable of binding and accumulating in healthy tissues, leading to an essential increase in the absorbed dose in healthy organs. For this reason, we tried to observe investigations on effective and convenient ^212^Pb chelator development as well as preclinical and clinical trials with ^212^Pb-based bioconjugates for radionuclide therapy of malignant neoplasms.

**Figure 2:**
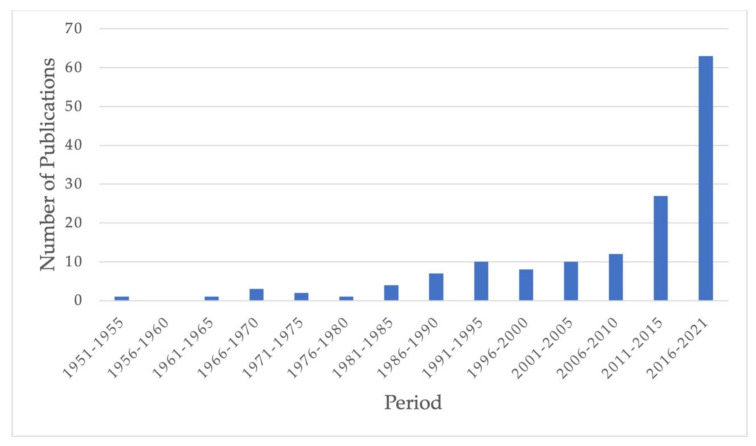
Dynamics of publications concerning ^212^Pb and its production for nuclear medicine (including reviews).

Below, we also present the dynamics of publications related to ^212^Pb use in nuclear medicine, including generators investigations papers, and reviews (Figure 2). Articles indexed in Web of Science and Scopus were considered. We can see that fast-growing interest in this radionuclide exists, especially during the last decade. This circumstance may indicate encouraging results of recent investigations, which are shown further.

## 2. Primary Production Techniques from the Beginning to the Present

The first concepts of ^228^Th descendants’ separation were reported in the 1930s [5]. It was discovered that barium salts of fatty acids demonstrated extremely high emanating power for radon and thoron [6]. A dry material with an emanation coefficient close to 100% appeared as a convenient source for ^220^Rn isolation and following ^212^Pb obtaining via its decay in a separate volume. In [7], the effects of gas pressure and source layer thickness were explored. The thickness of dry gas sources represents the key parameter of the emanation coefficient of source suspended matter; thus, obviously, emanating power increases with layer thinning. An attempt to develop a universal thoron source to yield its short-lived daughters for useful purposes was made in [6]. The authors reached the precipitation of source [^228^Th][Ba(C_18_H_5_O_2_)] powder with 99.5% of chemical yield at room temperature. The thoron source was placed in an open-ended column and deposited between two Teflon disks with a pore diameter of 9 µm. When room temperature air passed through this thin, dry thoron source, the release was greater than 95%. This type of source was also used in the investigations of aerosol particle formation with ^220^Rn progeny ions [8,9]. The principle of gaseous ^220^Rn carrying out for further capture was also realized in [10]. Natural thorium hydroxide powder served as a steady source of ^220^Rn for investigations on its progeny diffusion coefficients in various humidity values. Argon or clear room air was let into the reservoir with the source, thus moving ^220^Rn particles from the source powder to the particle detector.

Another approach was based on collection via electrodeposition and adsorption of derivatives. In [11], positive and negative plates were placed in a one-liter bottle; on the lower, positive aluminum plate, ^228^Th hydroxide was placed. ^228^Th hydroxide was obtained via precipitation from thorium nitrate solution. The potential between the positive plate and negative platinum coil caused positively charged ^212^Pb-ions to move to the negative plate, dissolving with, e.g., 3 M nitric acid. The total yield of the facility was 20%, so other methods of efficient ^212^Pb production were required. Moreover, the issue of ^224^Ra presence on the coil’s surface was not clarified. A remarkable approach was introduced in [12]. Namely, chemically purified, carrier-free ^232^U was electrostatically deposited on a steel plate and stored for 4 years to accumulate daughter nuclides. After placing this source at a distance of 0.2 mm from a separate material, the latter began to accumulate recoil nuclei of ^232^U daughters for further applications. After irradiation of the recipient material in a vacuum chamber, the alpha-spectrometric analysis showed that ^232^U-peaks were not observed on the target surfaces and proved that all activities were transferred by the recoil effect. Thus, this approach could not be used for pure ^212^Pb production.

Finally, we should broadly describe generator systems for ^212^Pb separation for radionuclide therapy investigations. Nuclear medicine generator systems typically consist of a long-lived parent radionuclide, with a T_1/2_ of several days to months, absorbed on a chromatographic material, whereas daughter radionuclide is periodically eluted with a small volume of solution [13,14]. In [15], ^228^Th solution in 3 M HNO_3_ was used to develop a generator for carrier-free ^224^Ra production. After evaporation, the residue was dissolved with 0.1 M HCl and then passed through Chromosorb W resin, followed by further ^224^Ra elution with the highest yield of approximately 75%. A novel approach to ^212^Pb production was presented by Zucchini and Friedman [16]. Here, the generator consisted of two major parts, a column with sodium titanate absorbent and a second column with D-50 cation exchange resin. Both ^228^Th and ^224^Ra (at levels up to 37 MBq) were absorbed into Na_2_TiO_3_ via passing thorium and radium containing dilute HCl (pH 6.0). Elution of ^220^Rn was conducted with H_2_O followed by ^212^Pb absorption in the second column. ^212^Pb could then be eluted with 2 M HCl with a maximum yield of 85%. Unfortunately, problems occurred when the system was scaled up to hundreds of MBq levels. Mainly, greater pressure increased the probability of leaks and subsequent floods, including thorium leakage further down the system.

For this reason, the authors developed an improved ^212^Pb generator [17]. It uses ^224^Ra with its much shorter (3.6 days) half-life as the parent source. The separation of radium and thorium was conducted earlier with an anion exchange resin after forming a thorium nitrate anion in concentrated HNO_3_. After double percolation of thorium-containing solution through the exchange column, it was evaporated, and radium with its descendants was picked up by 0.1 M HCl solution for binding to the generator. The key unit of the generator is a strong cation exchange resin column. The generator could be eluted with 0.5–2 M HCl or 0.2–2.0 M HI with yields of 50% and 90% for ^212^Bi and ^212^Pb, respectively. Concerning radionuclide purity, the breakthrough of parent ^224^Ra is less than 1 ppm in the case of the «fresh» generator, while the leakage of the «spent» due to radiolysis was also less than 0.04%. This ^224^Ra/^212^Pb-generator was used for investigations with desired radionuclides for more than 30 years; in other words, actual ^212^Pb production is based on this ^224^Ra/^212^Pb-generator, with levels of ^212^Pb activity up to ~600 MBq (16 mCi) [18,19,20,21,22,23,24]. Nowadays, it is supplied mainly by OranoMed and Oak Ridge National Laboratory [25,26,27,28]. Actual implementation involves an ion exchange separation of ^228^Th from ^232^U followed by a separation of ^224^Ra from ^228^Th with subsequent absorption of ^224^Ra in strong cation exchange (e.g., MP50) column.

While the ^224^Ra/^212^Pb-generator authors offered a procedure for obtaining an ultraclean solution for contemplated reactions with chelators, researchers [29] suggested a convenient approach to remove any traces from the final ^224^Ra/^212^Pb-generator solution. Eluate was moved through a column packed with Dowex-50X8-100 cation exchange resin to remove residual ^224^Ra, then, the effluent was passed through a Dowex-1X-200 anion exchange column to let all initial resin debris and fragments leave with this eluate, and finally to elute ^212^Pb from this anion exchange column with 2 M HCl for further processing. Horwitz, with colleagues [30], presented a technique called Multicolumn Selectivity Inversion Generator for radionuclides. A double cascade of two sequent chromatographic columns provided a highly pure daughter solution. After passing the initial parent and daughter solution through the daughter-selective column, the strip solution with daughter ions passed further through the parent-selective one for additional purification. For example, 0.1 M HCl with ^224^Ra and its daughter radionuclides was primarily percolated through LN-2 extraction resin to remove ^224^Ra/^212^Pb followed by moving ^212^Bi in 1 M HCl through strong acid cation exchange to remove residual parents from ^212^Bi. Using this technique, removal of ^224^Ra/^212^Pb from ^212^Bi by an overall factor of >10^8^ was achieved. Obviously, double cascade could be provided for ^212^Pb elution with corresponding ion-exchange resins. Later, in [31], a generator for ^212^Pb and ^224^Ra from ^228^Th was developed. A 4 M HNO_3_ combination preconditioned with UTEVA resin, Sr resin, and Prefilter resin (to adsorb traces of extractant) was loaded with a solution of ^228^Th with progeny. ^228^Th is retained by the UTEVA resin, and ^212^Pb is retained on the Sr Resin, while ^224^Ra passes through the three cartridges unretained. ^212^Pb and ^228^Th could then be recovered with yields of >95% and 99%, respectively, and Pb with <0.001% Th/Ra impurity. This generator was tested with ^227^Th, ^223^Ra, and ^211^Pb surrogates, so it will be of interest for ^212^Pb production with high radionuclide purity.

Another approach for ^212^Pb separation from carrier-free ^224^Ra was developed by Narbutt et al. [32]. In order to build up a reasonable amount of carrier-free ^228^Th in a separated ^228^Ra fraction, one must wait a year or more. On the other hand, ^232^Th in equilibrium with ^228^Th can also be the source of ^224^Ra and its descendants. For this reason, a 30-year-old Th(NO_3_)_4_·4H_2_O compound was used to separate ^224^Ra from natural thorium nitrate via solvent extraction with HDEHP instead of ion exchange due to low specific ^232^Th activity and thus extremely high ion-exchange resin volume. After following radium absorption from the aqueous phase on cation exchange resin DOWEX 50WX8, its progeny could be eluted with 0.5 M HCl (^212^Bi) or 1 M HCl (^212^Pb). The number of radionuclide impurities was less than 0.02%. Unfortunately, this approach was initially limited by natural thorium-specific activity, e.g., for 37 MBq (1 mCi), obtaining one must utilize more than 9 kg of ^232^Th with this approach.

Another concept was developed and described by Boll et al. [33]. An installation of several consequent ion-exchange columns was used to separate carrier-free Ra radioisotopes and carrier-free ^225^Ac from macro amounts of thorium via a several-step elution process. A mixture of thorium isotopes with its daughters was passed through the system of MP1 anion-exchange resin columns to remove Th-complexes, followed by further effluent passing through columns with cation-exchange AG50-X4 to separate ^224^Ra/^225^Ra from ^225^Ac. The work is of interest due to the presence of a coherent description of thorium decay series radionuclides chemical separation to provide a final product of high chemical and radioisotope purity. The two sequential MP1 columns provided a separation factor of 10^6^ for Ra/Ac separation from Th, and the two sequential AG50 columns were approximately 10^2^ for Ac from Ra. These ^224^Ra/^225^Ra could serve as parent source for obtaining ^212^Pb. This was applied for practical purposes in [34], where ^212^Pb ions were allowed to recoil into C_60_ to form ^212^Pb@C_60_ and its derivatives for further applications in radioimmunotherapy.

The USA Department of Defense transferred ~11 GBq of ^232^U to Alphamed Inc. from stocks at the Oak Ridge National Laboratory, and OranoMed reached success in the extraction of ^212^Pb from natural thorium salt [35]. Successive separation, purification, and concentration of ^232^Th/^232^U descendants resulted in the practically unlimited supply of ^212^Pb. It also should be noted that ^212^Pb(HNO_3_)_2_ is commercially available from OranoMed [36].

From 2010, nuclear medicine applications of ^212^Pb and relative radionuclides continued to be offered. A prospective anti-tumor approach called DART (diffusing alpha-emitters radiation therapy) was recently developed [37]. After ^228^Th is applied to a silicon surface via an evaporating HCl ^228^Th-containing solution, its progeny ^224^Ra recoil atoms leave (20–30%) the surface and, due to potential existence, reach stainless steel acupuncture needles. Despite the relatively low ^224^Ra desorption probability, the fraction of atoms reaching the needles is more than 95%, with a 5–15 mm distance between the ^224^Ra source and receiver. Then, these needles are used for anti-tumor procedures according to brachytherapy protocols.

Despite the advantages of the described generators, various other types of generators based on the separation of gaseous ^220^Rn from parent ^228^Th have since been developed. The first remarkable approach was proposed in [38] based on the aforementioned high emanating coefficient of barium stearate salt. According to this procedure for the synthesis of dry nitrate, ^228^Th was used for [^228^Th][Ba(C_18_H_5_O_2_)] [6]. After precipitation of the desired powder, it was wrapped up in special membrane filters, forming «packets». The essence of the study was to position source packets in the volume of the collection chamber to deposit the desired ^220^Rn decay products on its walls. The obvious advantage of this generator compared to those based on ionic exchange is the cost of operation. However, the yield of ^212^Pb production at activities 40–50 MBq decreased from 50% to 11% for 1 year due to radiolytically damage of dry ^228^Th absorbent. In order to avoid the destructive effects of radiolysis, an attempt to construct a low radioactivity-to-mass ratio source was made [39]. It was based on the concept of collecting ^220^Rn emanating from [^232^Th/^228^Th][Ba(C_18_H_5_O_2_)] source and consisted of two primary compartments, ^228^Th source and the ^220^Rn collector. The source was folded in thin layers inside a certain cage. Dry air was passed continuously through it, carrying gaseous ^220^Rn from the cage into a glass bubbler, which served as ^212^Pb collector, with methanol or n-hexane at temperatures below the point of ^220^Rn condensation. After its condensation, the required decay product ^212^Pb was isolated and then could be recovered with HNO_3_. The recovered yield of the facility was approximately 70%. Although it could theoretically provide ^212^Pb up to 44.4 GBq activity, there are some issues with convenience for personnel and the safety of this facility.

Another concept of such ^212^Pb production for nuclear medicine investigations was implemented in [40] and used in [41,42,43]. A ^212^Pb generator with ^228^Th as a parent isotope was designed. The generator operation principle is based on transport by the airflow of gaseous ^220^Rn radionuclide emanating from strong base anion exchange resin containing ^228^Th into a helix-shaped collector vessel where post-decay ^212^Pb is collected and deposited on the collector walls. After a 72-h operation cycle of the generator, sampling of ^212^Pb in the form of a solution in 0.1 M HCl is executed with approximately 30% yield of ^212^Pb. The generator is reloaded once every few years. The generator is intended for medical, biological, and radiochemical investigations in the field of developing antineoplastic radiopharmaceuticals for targeted delivery. We should note that there are a number of investigations with ^228^Th progeny demonstrating its benefit in other practical fields [44,45].

## 3. ^212^Pb Radiochemistry and Applications

### 3.1. Chemical Characteristics

At ambient conditions, lead’s cation in aqueous solutions is characterized by a 2+ oxidation state. According to [46], the ionic radius of Pb^2+^ is 1.19 Å (coordination number (CN)6). Pb^2+^ is a borderline cation in terms of Pearson’s HSAB theory [47].

**Figure 3:**
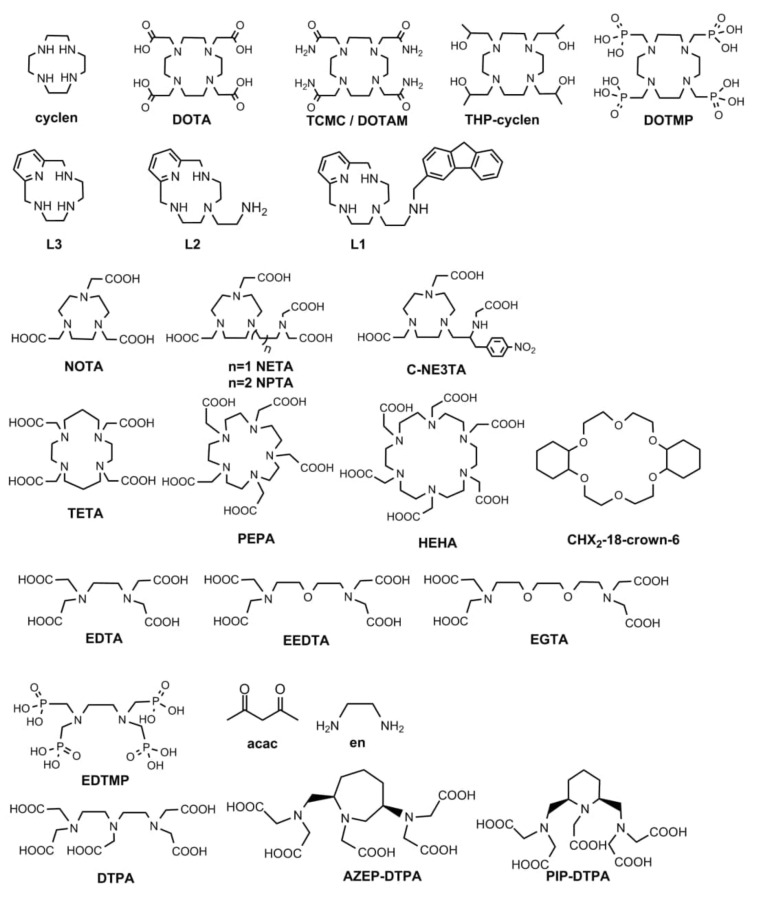
Ligands discussed in this paper.

The presence of the lone electron pair (LP) 6s^2^ in the electron shell of Pb^2+^ causes, in some cases, asymmetric coordination. The stereochemical activity of LP can appear in different degrees and has been debated for a long time [48,49,50,51,52,53]. In addition to the classical consideration of bonds’ lengths with the closest donor atoms in complexes with polyamines due to the X-ray single-crystal analysis [48,49], various quantum chemical calculations to reveal transition 6s^2^-6sp [51] or localize excessive electron density in Pb^2+^’s coordination environment [53] were performed. One of the major empirical conclusions in many works is that high CNs of ligand (>8) provide the lack of activity of LP (holodirected), while in complexes with low CNs (<6), LP is well pronounced (hemidirected). Namely, for smaller ligands, such as EDTA and ACAC [49,50], an obvious space in the first coordination sphere is observed. The latter is taken as a direct indication of hemidirected character. Other ligands such as 18-crown-6, CHX_2_-18-crown-6 [54], and EGTA [51] allow chelation of cations with longer interatomic distances where the basic atoms are distant from each other and correspondingly cannot crowd together on one side of a cation upon its coordination. In these complexes, Pb–L bonds are longer and differ less between each other, very likely indicating LP’s inactivity and uniform distribution of electron density of LP around the cation with regard to the distant location of donor atoms. In the frame of such consideration, holodirected or hemidirected behavior of LP in lead complexes with TCMC and DOTA are still highly controversial. Hereinafter, all ligands discussed through the paper are shown in Figure 3.

Possessing CN8 and the restricted close localization of donor atoms results in bonds’ asymmetry, e.g., Pb–O longer than Pb–N (Table 1); that is not the case for DOTA–chelates of other cations such as REE (Rare Earth Elements) or Sr^2+^.

Alternatively, completely unsubstituted cyclen (CN4) [49] and its pyridine analog (CN4) L3 [52] coordinate Pb^2+^ so as to leave the space for LP, the addition of one coordinating pendant arm in L1 [52] still allows one-side coordination with shortened Pb–N bonds on the side opposite to LP. Further introduction of four groups, TCMC, DOTA, THP-cyclen, causes a significant decrease in differences between values of bonds’ lengths Pb–N and Pb–O (Table 1). Moreover, from Pb–cyclen to Pb–THP–12aneN4 and from Pb–L3 to Pb–L2 [52], a small but regular decrease in logβ (PbL) occurs (Table 2). Such an effect is caused by the transition from hemidirected to holodirected LP [49,55] being thermodynamically disadvantageous. Similarly, in complex with EDTA LP is hemidirected, but upon ligand’s modification to EEDTA and EGTA LP of Pb^2+^ becomes inactive, leading to a large drop of logβ, while analogous modification does not cause such drastic break for Sr-EDTA and Sr-EGTA (Sr^2+^ and Pb^2+^ are similar regarding ionic radii [46]). Furthermore, basic nitrogen atoms bring covalent impact to the interaction with Pb^2+^ enhancing LP’s activity. This induces logβ drop upon substitution of NH-groups in cyclen by O-atoms [49]. However, accompaniment of N-donors by O-containing pendant groups increases the stability of formed complexes Pb–cyclen–Pb–DOTA or Pb–TCMC, Pb–en–Pb–EDTA.

The close ionic radii and oxidation state of Pb^2+^ and Sr^2+^ make the former toxic, especially for bones. It is well-known that poisoning by free lead cations affects its accumulation in the skeleton. Moreover, because it has an oxidation state of 2+, it can replace Zn^2+^ in the biosynthesis of heme, causing anemia.

### 3.2. Ligands and Other Carriers for Pb^2+^

Chelators such as EDTA and DTPA were actively studied for clearance of Pb^2+^ from organisms after poisoning [60]. It was shown that both act with similar efficacy, which agrees with one order of β (Pb–L) (Table 2).

To the best of our knowledge, DTPA was not evaluated for the chelation of ^212^Pb in target radiopharmaceuticals. However, modifications of DTPA were considered for ^212^Pb chelation for nuclear medicine [61]. Being flexible, acyclic ligand DTPA was rigidified via cyclization of the main backbone, producing AZEP-DTPA and PIP-DTPA. Complexes of ^203^Pb^2+^ with both ligands have demonstrated high stability in vitro in the presence of serum proteins, which proves the positive influence of cyclic fragments introduction into DTPA. However, in vivo experiments performed in normal mice with piperidine derivative (PIP-DTPA) showed quicker pharmacokinetics, indicating fast clearance and indirectly revealing in vivo stability in contrast to AZEP-DTPA. Furthermore, in order to evaluate the applicability of ligands for ^212^Pb chelation in radiopharmaceuticals, it is expedient to study the binding of these ligands with daughter radionuclide ^212^Bi. In bismuth complexes, the effectivity of PIP-DTPA is more pronounced than in lead complexes; upon injection of Bi–AZEP–DTPA, kidneys retained 5–10 fold higher levels of radioactivity than upon Bi–PIP–DTPA application.

**Figure 4:**
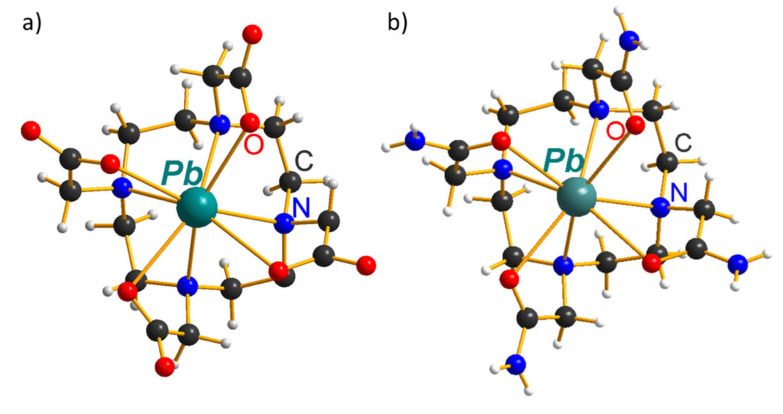
Crystal structure of: (**a**) Pb–DOTA; (**b**) Pb–TCMC complexes.

Currently, only two chelators were studied in detail for ^212^Pb chelation, DOTA and TCMC (DOTAM) [62], including immunotherapeutics based on mAbs (monoclonal antibodies) that are heat-sensitive (Table 3). Both these ligands are characterized by a low complexation rate at room temperature (rt), and formed complexes slowly dissociate, i.e., are inert. As soon as ligands are structurally close, both complexes form analogous crystal structures (Figure 4) and logβ values (Table 2) despite significant variation in affinity to protons pKa (DOTA) = 28 [63] vs. pKa (TCMC) = 14.86 [50]. In both structures, the coordinating polyhedron is a tetragonal antiprism formed by cyclen’s nitrogen and oxygens of acetic/acetamide groups [50,51].

However, direct comparisons of conjugates DOTA–mAb and TCMC–mAb have shown that the latter binds cation more effectively [64] (Table 3). Long manipulations with eluate from ^224^Ra/^212^Pb as well as synthesis and purification procedures of labeled compounds are possible due to the long half-life of ^212^Pb. It is noteworthy that in an exhaustive majority, synthesis at 37 °C for 30–60 min requires the purification via exclusion chromatography from unbound cation that was preliminarily chelated by DTPA or EDTA. This is a necessary step because of incomplete cation binding at low temperatures.

**Table 3:** Radiolabeling of monoclonal antibodies, peptides, and other molecules with ^203,212^Pb.

Conjugate	T °C, Duration	Purification from Free Pb^2+^	References
DOTA–AE1	35 °C, 45 min	EDTA–SE HPLC	[65]
TCMC–trastuzumab	37 °C, 30 min	EDTA–SE HPLC	[66,67,68]
	37 °C, 1 h		[69]
TCMC–cetuximab	37 °C, 1 h	EDTA–SE HPLC	[70]
TCMC–PSMA	95 °C, 5 min	-	[71]
TCMC–VCAM1	37 °C, 30 min	-	[36]
TCMC–CC49 (mAb)	37 °C, 30 min	EDTA–SE HPLC	[64]
TCMC–daratumumab	37 °C, 15 min	-	[72]
TCMC–TATE (DOTAMTATE)	50 °C, 10 min	-	[73]
DOTA–CC49 (mAb)	37 °C, 30 min	EDTA–SE HPLC	[64]
DOTA–Re(Arg11)CCMSH (mAb)	75 °C, 45 min	RP–HPLC	[24,74]
DOTATOC	85 °C, 45 min	-	[19]
DOTA–103A (mAb)	37 °C 30 min	EDTA–SE HPLC	[75]
DOTA–biotin	80 °C 30 min	-	[76]

Beta-decay of ^212^Pb accompanied by electron conversion yields the highly ionized state of a daughter atom of ^212^Bi. The latter provides Bi–L bonds’ breaks in complexes with DOTA [77] and release of ~36% of ^212^Bi from the complex, finding confirmation in many in vivo experiments [75,76,77]. A deviation from the equilibrium ratio of A(^212^Pb)/A(^212^Bi) is observed. This is especially true in the case of kidneys, a characteristic organ for unbound Bi^3+^ retention. A comparison of the biodistribution of the different isotopes ^212^Pb, ^203^Pb, and ^205/6^Bi [75,76] was carried out. As a result, increased ^212^Bi content was detected in kidneys upon injection of ^212^Pb complex and correspondingly unbound cation in urine, while with ^203^Pb and ^205/6^Bi, accumulation of Pb^2+^ or Bi^3+^ in kidneys was not detected, and only complexed forms of radioactivity were found in urine. This proves the influence of ^212^Pb’s decay on the fate of daughter ^212^Bi. Moreover, even after incubation of [^212^Pb]Pb–DOTA–biotin in an aqueous solution for 4 h, detection of ~30% of unbound (apparently released) bismuth cation is possible [76]. Concerning the [^212^Pb]Pb–TCMC complex, it was mentioned that 16% of ^212^Bi is released [78]. Moreover, by this time, a mathematical model for predicting the possible release of daughter radionuclide was developed and experimentally tested [79]. However, recently, PSMA ligands were labeled with ^212^Pb via bifunctional DOTA and TCMC [80], and a comparison of biodistribution of ^212^Pb and ^212^Bi did not reveal differences.

Regarding the large difference in the basicity of DOTA and TCMC, a comparison of the role of the medium’s acidity on the stability of complexes with Pb^2+^ is important. It was shown that upon the incubation of labeled mAbs’ conjugates [^203^Pb]Pb–DOTA–CC49 and [^203^Pb]Pb–TCMC–CC49 in the solutions with varied pH, the half-dissociation time for the former is 10–15 h at pH 2 and 150 h for the latter [64]. According to [57], the acid-catalyzed dissociation of Pb–DOTA begins from the protonation of the carboxylic group, and then this proton migrates onto macrocyclic nitrogen. Less basic acetamide groups of TCMC compared to acetic in DOTA provide lesser affinity to protons at the beginning, leading to Pb–TCMC complex inertness in an acidic medium [64,81]. However, both complexes demonstrated similar stability in the presence of competing chelating agents: 100% after 1 d. Further, the cation is released from both but from Pb–DOTA at a higher rate [64]. 

Complexes with other azacrown ethers NOTA and TETA were tested for stability in an acidic medium [82]. Both complexes were shown to dissociate in contrast to Pb–DOTA. In addition, the synthesis of [^212^Pb]Pb–NOTA was completely unsuccessful, with <10% chelated at pH6 [13]. In general, NOTA, being the smallest among azacrowns, is more appropriate for cations of a smaller radius such as Cu^2+^ (*R(CN6)* = 0.73 Å) or Ga^3+^ (*R(CN6)* = 0.62 Å vs. 1.19 Å for Pb^2+^ [46]) [83]. That is why hampered complexation of Pb^2+^ as well as low stability are coherent. Moreover, replacement of one acetic arm by ethylene-aminodiacetic (NETA) improves the stability in vitro; when challenged with serum proteins, Pb–NETA keeps 100% intactness in 11 days; and in vivo, fast clearance and the lack of retention in any organs [61]. The significance of the ethylene-aminodiacetic arm in NETA for chelation of Pb^2+^ was shown upon ineffective modifications: its elongation till propylene-aminodiacetic pendant arm (NPTA) [61] or bifunctionalization with Bn-SCN-moiety (C-NE3TA) [84]. The stability of both Pb–NPTA and Pb–NE3TA dropped in vitro [84] as well as in vivo [61]. Due to the lack of structural data for the discussed complexes, one can suggest that dislocation of coordinating sites in NETA provides the most effective chelation of Pb^2+^ and any change, such as an increase in the distance between them or decrease in the stereochemical availability via bulky Bn-SCN bifunctional group crucially affects the stability of the complexes formed.

Other cases of complexation of Pb^2+^ by azacrown-ethers DOTA, PEPA, and HEHA were suggested for analytical purposes, namely, determining the number of chelator molecules per protein molecule after conjugation [85]. However, it was not the case for TETA as a result of low logβ (Pb–TETA), causing the lack of competition with Arsenazo III for Pb^2+^.

Another related class of organic compounds evaluated for application as part of radiotherapeutics with ^212^Pb is phosphonate ligands, including other functionalized cyclen DOTMP and EDTA analog EDTMP. Similar to [^212^Pb]Pb–DOTA complex, [^212^Pb]Pb–DOTMP releases ^212^Bi upon the decay of ^212^Pb, leading to renal accumulation of freed bismuth, while no released of ^212^Bi were found when [^212^Bi]Bi–DOTMP was injected [86]. However, both bismuth and lead complexes with DOTMP demonstrated higher stability in vivo compared to chelates with acyclic ligand EDTMP [86].

Besides organic ligands, other carriers, such as liposomes [87] and fullerenes [34], were also considered. Such kinds of transporters can provide the absence of release of daughter radionuclides after alpha decay due to recoil. On the other hand, the recoil of the ^212^Pb nucleus from ^224^Ra was used to incorporate ^212^Pb into fullerenes (C60) for the synthesis of labeled fullerenes ^212^. It is of interest that Pb@C60 [34] also had ^213^Bi originating from ^225^Ra that was present as an impurity in ^224^Ra, while no ^225^Ac was found. The latter was caused by a lack of recoil in the decay of ^225^Ra but sufficient upon formation of ^213^Bi. The feasibility of this labeling path was demonstrated, but the yields did not exceed 1%. Despite no activity being detected in bones, meaning stability of ^212^Pb@C60 regarding dissociation in a live organism, modification of fullerenes is required since in vivo experiments revealed retention in the spleen and liver.

### 3.3. Preclinical, Animal Studies

Among earlier works regarding the therapeutic application of ^212^Pb, a series of papers [88,89,90,91] was dedicated to the assessment of various forms labeled by ^212^Pb: labeled liposomes [91], sulfur colloids [90], and colloids of iron’s hydroxides [89]. Cell lines of ovarian cancer and xenografted mice with epithelial ovarian and Erlich’s cancer for in vivo tests were used. In this case, an intraperitoneal (I.P.) injection was used to study the character of abdominal tumors. The non-uniform distribution of colloids caused by the inappropriate size of colloids led to the sorption of radioactivity on the colon and corresponding necrotic lesions. The latter caused adverse effects on the gastrointestinal system and bone marrow. Although ^212^Pb was injected without targeting moiety, only chelated by DTPA study of cytotoxicity in vitro revealed dose-depending burdens of cells’ chromosomes [89], meaning that even the presence of ^212^Pb in the surrounding medium matters. In addition, 15 and 50 µCi (555 kBq–1850 kBq) in the form of [^212^Pb]Fe(OH)_2_ were effective for tripling the survival period of mice, whereas 24% had complete remission.

The majority of the further research in this field was carried out with targeting molecules: peptides and monoclonal antibodies (mAbs) that possess selectivity for definite cells. To safely apply highly toxic alpha-radiation, the determination of a range of applicable doses is required at the beginning [75].

The range of tolerant doses was estimated in various papers [28,65,75]. It was empirically shown that the effectiveness of radioimmunotherapy (RIT) by ^212^Pb depends on the size and/or age of the tumor xenografted. The normal mice, as well as mice implanted with erythroleukemia [75] and ovarian cancer [65], were treated by respective antibodies labeled by ^212^Pb (Table 3). The activity levels of 20 [75] and 40 µCi [65] (740 and 1480 kBq) were found to induce fatal marrow toxicity in normal mice. Nevertheless, 10–20 µCi (370 and 740 kBq) caused complete remission for smaller size tumors if the therapy was started on the third day after cancer cells’ inoculation [65]. Other cases of treatment initiation on the 8–14th day [75] and later when tumor volume reaches >15 mm^3^ [65] yielded only a partial decrease in its size. Moreover, for large burdens of 146 mm^3^, no effect was detected. Apparently, this was caused by the lack of crossfire effect for alpha-emission compared to beta-emission, causing the therapy of large solid tumors by ^212^Pb to be ineffective. Interestingly, the presence of β-particles (*E(β_max_)* = 574 keV) from ^212^Pb itself did not impact the crossfire effect. It could be the sequence of the quantity of the applied activity of ^212^Pb compared to GBq levels of activities for traditional β-emitting radiopharmaceuticals with ^177^Lu (*E(β_max_)* = 498 keV) and ^90^Y (*E(β_max_)* = 2280 keV) [92,93].

For in vivo mice experiments, only doses lower than 20–40 µCi (740–1480 kBq) were used [23,66,68,94,95,96]. However, in [24], for the treatment of palpable melanoma, 50–200 µCi (1850 kBq–7400 kBq) of ^212^Pb were applied. Renal toxicity for 100 and 200 µCi (3700 kBq and 7400 kBq) was revealed in histological tests of the kidney’s cortex, while no external symptoms were observed. Therefore, dose-limiting organs were kidneys, not bone marrow, compared to the aforementioned papers. This was related to the affinity of the antibody to kidneys. It is noteworthy that these 100 and 200 µCi (3700 kBq and 7400 kBq) were not only less toxic but also caused tumor-free survival with a 2–3-fold elongation of mean survival.

Monoclonal antibodies labeled with ^212^Pb were shown to be cleared without retention in organs, and, correspondingly, no irradiation of healthy tissues occurred. However, in the case of injection in xenografted mice, no significant myelotoxicity was found. Bone marrow toxicity was associated with metabolic processes following the internalization of mAb in malignant cells [75]. This degradation of mAb includes transportation to the liver, the second organ of radioactivity accumulation after bone. The latter retained Pb^2+^ after acid-mediated dissociation of [^212^Pb]Pb–DOTA–mAb during its degradation. That is why TCMC, being more stable in the acidic conditions complexes [64], was a preferred alternative for DOTA as part of ^212^Pb radiotherapeutics.

After conjugation of TCMC with trastuzumab, [^212^Pb]Pb–TCMC–trastuzumab was thoroughly studied [68,94,95,97,98,99]. One of the first detailed analyses of therapeutic efficacy of [^212^Pb]Pb–TCMC–trastuzumab towards two types of HER2-expressing tumors was performed in [68]. According to in vitro experiments with cell lines, the advantage of ^212^Pb over ^212,213^Bi was established. MTD was stated to be 20–40 µCi (740 kBq–1480 kBq) owing to survival and weight loss. This value agrees well with earlier published estimations [65,75]. Furthermore, even upon fractionated injection of 30–40 µCi (110 kBq–1480 kBq) by 10 µCi (370 kBq), these values led to a decrease in effectiveness and shortened survival [68]. The best results in single or fractionated injections were an extension of the survival period by 2–4 times depending on the dose injected. It is noteworthy that regarding previous results of [65], this therapeutic study was demonstrated on 3- and 5-day colon cancer tumors.

Further, this group studied the effects of combined application of chemotherapeutics such as gemcitabine [94] and paclitaxel [95] with [^212^Pb]Pb–TCMC–trastuzumab. Different schemes of chemo and radioimmunotherapeutics were varied in vivo, and analyses of the cell mechanisms managing their effects were attempted [20,97,98]. The research series described above [68] was carried out on a human colon carcinoma model in xenografted mice. The cells possess affinity towards Herceptin (commercial name of trastuzumab), and this cancer can form a disseminated peritoneal disease. It was shown in vivo that a combination of radiosensitizing gemcitabine [94] and paclitaxel [95] with target radiotherapy gives a synergistic effect, whereas such a therapeutic effect cannot be reached by these therapeutics separately and without targeting moiety. Moreover, it was shown that treatment is effective even with 5 and 10 µCi (180 and 360 kBq) of ^212^Pb. In [94], four methods of treatment were tested concerning the action of chemotherapeutics, along with the results of a previous method. The best results were achieved when four doses of gemcitabine (1 mg) and two doses (10 µCi, or 360 kBq each) of [^212^Pb]Pb–trastuzumab [94] or 0.6 mg paclitaxel and 10 µCi (360 kBq) of ^212^Pb [95] were applied in the definite order. These approaches provided ≥10 and 4 fold prolongation of median survival of xenografted mice compared to non-treated or treated with [^212^Pb]Pb–trastuzumab alone [94,95]. Tumor cell analysis revealed that upon injection of [^212^Pb]Pb–trastuzumab alone [97] or with paclitaxel [20] or gemcitabine [98,99], genes responsible for cell cycle arrest, apoptosis, and corresponding mechanisms leading to cell death or depression of successful reproduction were induced. Surprisingly, no induction of genes involved in DSB-repair was observed, while expression of genes repairing single-strand breaks was increased [97]. Moreover, in cases with [^212^Pb]Pb–trasuzumab alone or accompanied by paclitaxel, no recovery of the cell cycle was observed, while treatment with non-specifically targeted ^212^Pb was shown to cause repairable cell cycle [20].

Similar to TCMC–trastuzumab bioconjugate, DOTAMTATE (TCMC–TATE) was preclinically studied for therapy of neuroendocrine tumors (SSTR-positive) labeled with ^212^Pb [100] as a single treatment agent and in combination with chemotherapeutic 5-fluorouracil [73]. This study confirmed tolerated doses of 20–40 µCi (740 kBq–1480 kBq) of [^212^Pb]Pb–DOTAMTATE. Moreover, different regimes of fractionated injection of radioactivity with varied periods between injections showed that application of 3 × 15 µCi led to 100% survival of normal mice in contrast to 50% when 2 × 20 µCi and 1 × 40 µCi 30 weeks after starting of treatment due to significant hematological toxicity [71]. It was shown that combination of fractionated injection of [^212^Pb]Pb–DOTAMTATE (3 × 10 µCi) with 5-fluorouracil resulted in 79% of mice surviving vs. <50% for [^212^Pb]Pb–DOTAMTATE (3 × 10 µCi) alone after a 31-week period. It was caused by an effective slowdown of tumor growth without full depression because, at the beginning of therapy, the tumor achieved a significant size of 100–300 mm^3^.

Moreover, regarding the successful application of alpha-emitting ^225^Ac and ^213^Bi for the treatment of prostate cancer [101], PSMA ligand was also conjugated with DOTAM (TCMC) [18,80] and tested in vivo with ^212^Pb on xenografted models. Consequent elaboration of conjugates of PSMA-inhibiting moiety with TCMC was performed specifically for ^212^Pb [18,71,80,102]. The well-tolerated dose of 9 µCi (330 kBq), in agreement with the above-mentioned values, was shown to demonstrate survival elongation, inhibition of tumor growth, and the same therapeutic index as PSMA-617 labeled by ^225^Ac (0.5–2.7 µCi, 1850–9990 Bq) or ^177^Lu (54–3000 µCi, 200 kBq–11.1 MBq) [18]. This is due to the high dose rate delivered to the malignancy by ^212^Pb because of the lower half-life and correspondence of biological half-life of labeled conjugate and physical half-life of radionuclide.

In order to evaluate the role of the internalizing character of a targeting moiety, alpha radiation’s effectiveness was tested for small volume (10 mm^3^) peritoneal carcinomatosis [96,103] with two types of mAbs 35A7 (non-internalizing) and trastuzumab (internalizing). It was shown that, upon the injection of 40 µCi (1480 kBq), hematological toxicity appeared, while no renal or hepatic influence was detected. This value was crucial for bone marrow and kidneys with other mAbs in the aforementioned papers [65,75]. It is possible that, in addition to mAb replacement, immunoconjugates studied in [103] were prepared with TCMC instead of DOTA, which prevented dissociation and respective irradiation of dose-limiting organs. As a result, [^212^Pb]Pb–trastuzumab caused a higher increase in median survival for non-treated compared to [^212^Pb]Pb–35A7 despite a lower dose absorbed [96,103] from 18 d to >125 d vs. 94 d. This can be a sequence of synergistic interaction of trastuzumab itself accompanied by alpha-radiation, although individually trastuzumab did not affect this tumor [96].

Trastuzumab, labeled with ^212^Pb, was assessed for the treatment of prostate cancer [23,66]. In contrast to I.P. injection for abdominal cancer treatment [68,94,95], these experiments were performed using an intravenous (I.V.) injection of [^212^Pb]Pb–TCMC–trastuzumab. Neither hematological, histological, liver, or kidney toxicity nor significant weight loss in the time period of 21 days after treatment was observed [66]. However, biodistribution in 30 min at 3 days showed that spleen, liver, and kidney retained a surprisingly high percentage of ID [23]. This appears to correlate with moderate curative ability; the median survival was lengthened from 47 to 61 days with 20 µCi (740 kBq) of ^212^Pb [66]. This modest response could be caused by the low expression of HER2+ receptors by this cell line [66], leading to the long circulation of labeled antibodies in the blood pool and accumulation in organs with large blood supply [23]. On the other hand, low expression of specific receptors towards trastuzumab was supposed to be effective for treatment by alpha-radiation [68], but obviously, it depends on how low the expression is.

Besides trastuzumab, recently, ^212^Pb was demonstrated to be effective in preclinical studies for the treatment by labeled antibodies of multiple myeloma [72], non-Hodgkin lymphoma [104], ovarian cancer [105], and pancreatic adenocarcinoma [26].

Targeted therapy with ^212^Pb was also evaluated for the treatment of brain metastases [36,106]. Due to the blood–brain barrier, application of the suggested anti-VCAM1 (vascular cell adhesion molecule 1) antibody assumes irradiation of early brain metastases in the vicinity of vessels (depth ~50 µm), lacking the necessity to penetrate through the vessel wall. Starting from modeling by the Monte Carlo technique, it was shown that emission of ^212^Pb with two highly energetic alpha-particles in the decay chain could provide a high yield of double-strand breaks per decay in the 40–100 µm range [106,107]. The feasibility of this approach was experimentally approved for mice with brain metastases from breast cancer. The therapeutic effect and significant increase in overall survival along with lack of liver and blood toxicity were demonstrated [36]. Moreover, increased expression of the corresponding receptors was observed after external irradiation therapy causing enhancement of the effectiveness of [^212^Pb]Pb–VCAM1 treatment following the external irradiation.

Another approach for specific delivery of radioactivity can be the pretargeting technique when malignancies or other organs of interest are bound with an affine molecule that does not contain radioactivity. This process of binding pretargeting compounds with affected tissue can take a prolonged time. Then, labeled by radionuclide specific molecule is injected and quickly accumulates in the areas with the pretargeting units. This method was already tested with [^212^Pb]Pb–DOTA–biotin and streptavidin as the pretargeting molecule [76]. It was shown that upon injection of 10 µCi (360 kBq) in xenografted mice, high tumor/marrow and tumor/kidney ratios were observed, while in healthy mice, radioactivity cleared quickly. The latter proved the applicability of this concept, especially for ^212^Pb/^212^Bi, regarding their high bone marrow and kidney toxicity.

In addition to biomolecules and their synthetic analogs and derivatives, low molecular weight compounds bearing affinity to specific organs can be used for target delivery. Phosphonate derivatives of EDTMP and DOTMP [86] were tested for addressing radionuclides to tissues with an increased phosphorus intake upon the growth of bone malignancies. The [^212^Pb]Pb–DOTMP was shown to be quickly cleared from the blood pool and accumulated in bones in the frame of searching alpha-emitting alternative for radiopharmaceutical [^153^Sm]Sm-EDTMP [86] was used for therapy of bone malignancies and metastases. Furthermore, the application of ^224^Ra for the treatment of skeletal metastases and peritoneal malignancies is hindered by the uncertain fate of the relatively long-lived progeny radionuclide ^212^Pb [108,109]. According to its affinity towards EDTMP, this ligand can be added to applied solution or suspension to provide fast chelation of the released ^212^Pb. In the case of the ^224^Ra chloride salt solution, ^212^Pb chelated by phosphonate is returned to bone metastases for treatment along with ^224^Ra [109].

According to most preclinical studies discussed above, one of the main questions is the dose of ^212^Pb that can be safely used for therapy. That is why before clinical trials [^212^Pb], Pb–TCMC–trastuzumab was assessed in monkeys as soon as they had similar to human expression of Her2+ receptors [21]. Interestingly non-eo was found in the ratio of activities, ^212^Pb/^212^Bi = 1.1–1.5, on the first time points, which could be due to the loss of bismuth from the peritoneal cavity or caused by the inequality in the detection of 236 keV (^212^Pb) and 738 keV (^212^Bi) by the gamma camera. This did not affect any serious toxicity; no adverse effects except slight to bone marrow were observed. Even for bone marrow, no damage was detected upon microscopic evaluation of its cells. The lack of toxicity was promising since, for human experiments, twice lower doses of radioactivity were suggested [110,111].

Besides the direct empirical finding of tolerated doses in mice and other organisms, compounds labeled with ^203^Pb and SPECT can be used as a surrogate for ^212^Pb in some labeled peptides [19]. In order to evaluate the dosimetry of [^212^Pb]Pb–TCMC–PSMA, this ligand was labeled with ^203^Pb and tested by SPECT in patients accompanied by phantom application and calculations [71]. However, in this case, the biodistribution of radioactivity in the case of ^203^Pb cannot be simply translated for ^212^Pb compounds due to peculiarities of the latter’s decay, making the fate of daughter radionuclide ^212^Bi uncertain [77].

Additionally, it was shown that despite many daughter radionuclides and their emission, quantitative SPECT of ^212^Pb itself is possible [112]. The latter opens up prospects of theranostic application, dosimetric estimation, and evaluation of treatment feasibility by ^212^Pb radiopharmaceuticals in each case.

### 3.4. Clinical Study

The first clinical trials with ^212^Pb were performed from 2011 to 2015. The results are published [110,111,113]. A total of 18 patients with HER-2 expressing malignancies, ovarian and colon cancer, were treated intraperitoneally. Six doses in the range 0.2–0.74 mCi/m^2^ (7.4–27.4 MBq/m^2^) were tested. It was shown that upon dose-escalation, the disease stabilization (SD) had a more pronounced and prolonged character while no partial or complete response was shown [113]. SD peaked in 24 weeks post-treatment with 0.57 mCi/m^2^ (21.1 MBq/m^2^). However, all patients had progression of disease in <8 months. Almost all adverse effects except abdominal pain were related to accompanied medications or conditions as pre-treatment by trastuzumab, catheter, diuretics, SSKI (saturated solution of potassium iodide for protection of thyroid) [110,111], or earlier developed anemia [113]. The major conclusion of this trial was that even the highest dose among those studied did not cause serious hematological, renal, or liver toxicity indicating the possibility of increasing the dose and using this therapy in combination with other agents [113]. This low toxicity of ^212^Pb is associated with intraperitoneal injection accompanied by intravenous injection of blocking agent. According to dosimetric calculations [111], peritoneal fluid, among other parts of the body, possessed 200–300 mSv/MBq while others had two or more orders lower values, including the impact from ^212^Bi, ^212^Po, and ^208^Tl.

According to a successful preclinical study of [^212^Pb]Pb–DOTAMTATE (^212^Pb–AlphaMedix^TM^) for treatment of neuroendocrine tumors [73], clinical trials were initiated in 2018 and now are in progress [114]. As of today, the optimal dose was found, and a high Objective Radiological response of 83% compared to 13% of [^177^Lu]Lu–DOTATATE was shown [115].

## 4. Conclusions

In the last decade, it was shown that highly toxic alpha-emission could be exploited with more benefits than drawbacks. Among alpha-emitting radiometals according to availability, chelation chemistry, and half-life, ^212^Pb is optimal.

As it is a beta-emitter, ^212^Pb can provide alpha radiation as an in vivo generator of short-lived alpha-emitters ^212^Bi and ^212^Po. Two alpha-particles with high energy determine the therapeutic effect in targeted therapy of tumors as well as micrometastases, including brain metastases. Besides the overall restricted and cautious application of alpha-emission in radiotherapy, ^212^Pb has individual limitations. The high energy of gamma-emission of progeny radionuclide ^208^Tl (2.6 MeV, 99%) provides a high dosimetric load on a patient and medical personnel. That is why dosimetry and toxicity are accurately evaluated in the frame of preclinical and clinical studies. Finally, the optimal dose for the treatment of neuroendocrine tumors, colon and ovarian cancers can be established. Therefore, the solution to the only significant remaining drawback to using ^212^Pb in RIT, high-energy γ-ray emitted by the ^208^Tl daughter, namely, shielding healthcare personnel from high-energy γ-rays, is well known and justified due to the ^212^Pb encouraging results in therapy.

Another point is the release of 36% daughter ^212^Bi from the [^212^Pb]Pb–DOTA chelate, causing uncertain behavior of alpha-emitting progeny radionuclides in the living organism and irradiation of non-target tissues. Currently, the problem is being solved by using tetraamide cyclen (TCMC) for chelation of ^212^Pb in the radiopharmaceuticals instead of DOTA. Due to boundary hardness and divalent nature of Pb^2+^ 12-azacrown-4 tetraamide derivative (TCMC, DOTAM) strongly binds the Pb^2+^. The TCMC is one of the main ligands for the preclinical and clinical evaluation of ^212^Pb–radiopharmaceuticals requiring bifunctional chelators. The development of biomolecule, its clearance, and internalization inside the cancer cells can minimize the toxic effect of the release of ^212^Bi from the ^212^Pb–chelate.

Due to a half-life of 10.64 h, ^212^Pb can be successfully applied with biovectors bearing different pharmacokinetics, including monoclonal antibodies, peptides, and molecules of pretargeting approach for the treatment of various malignancies. Many biological molecules as antibodies and peptides were labeled with ^212^Pb and tested preclinically to estimate the dosimetry and therapeutic ability. It is noteworthy that as soon as the application of alpha-emitting ^224^Ra is restricted due to the formation of relatively long-lived ^212^Pb and lack of chelators for Ra^2+^ cation, the chelates of ^212^Pb as representative of the decay chain of ^224^Ra can be used alternatively but obviously with twice fewer alpha-particles per decay. Moreover, the ^212^Pb/^212^Bi in vivo generator delivers a dose per unit of administered activity 10 times greater than in the case of ^212^Bi alone or of the ^213^Bi α-emitter [13]. Finally, ^225^Ac and ^212^Pb show a similar capacity for inducing DNA damage per unit absorbed dose [107]. Additionally, compared to other alpha-emitting radiometals such as ^225^Ac/^213^Bi and ^212^Bi, it is obvious that the half-life of ^212^Pb appears to be the most suitable from dosimetric and radiosynthetic points of view. Regarding ^149^Tb, the supply of ^212^Pb looks much more sufficient and reliable for preclinical and clinical studies than obtaining of ^149^Tb at the present time. We also believe that ^212^Pb could be more useful in some cases when the decay rate plays a key role in treating fast-growing tumors.

Concerning the supply of ^212^Pb, pharmaceutical companies developed innovative methods for α-emitter production, with practically unlimited capacity to produce high-purity ^212^Pb, so that high cost and limited availability have almost been overcome. Moreover, the use of elementally identical ^203^Pb with therapeutic ^212^Pb represents an encouraging theragnostic pair due to the favorable ^203^Pb half-life (T_1/2_ = 52 h) and decay, with 80.1% emission of γ-rays at 279 keV, so it is compatible with single-photon emission computerized tomography (SPECT). This makes ^203^Pb ideally suited as a matched radionuclide tracer for ^212^Pb to eliminate the execution of accurate biodistribution and targeting assays of ^212^Pb–radiolabeled molecules, as has already been declared [116].

In summary, the study of this radionuclide as a radiotherapeutic agent has been significantly facilitated in the last years in the frame of development of alpha-therapy in general. This prospective therapeutic radionuclide considerably widens the range of alpha-emitting radionuclides suitable and available for the implementation and clinical application of targeted alpha-therapy in various approaches.

## Figures and Tables

**Table 1:** Bond lengths in crystal structures of Pb2+ complexes with polyamines.

Compound	Pb–DOTA [51]	Pb–TCMC [48]	Pb–TCMC [50]	Pb–THP–Cyclen [55]	Pb–L3 [52]	Pb–L1 [52]
Pb–N1	2.687	2.654	2.617	2.64	2.4	2.53
Pb–N2	2.638	2.61	2.625	2.64	2.524	2.64
Pb–N3	2.66	2.641	2.628	2.64	2.42	2.51
Pb–N4	2.676	2.629	2.63	2.64	2.502	2.59
Pb–O1	2.796	2.777	2.657	2.71	3.11	3.03
Pb–O2	2.856	2.902	2.785	2.71	3.22	3.09
Pb–O3	2.827	2.667	2.756	2.78	2.96	-
Pb–O4	2.609	2.77	2.819	2.78	3.11	-

**Table 2:** Stability constants for some Pb–L complexes.

Ligand	Cyclen	DOTA	TCMC	THP-Cyclen	NOTA	TETA	L3 [52]
logβ	15.9 [49]	22.7 [56] 24.3 [57]	>19	15.07	16.6 [57]	14.3 [56]	15.71
Ligand	en	EDTA	EEDTA	EGTA	DTPA	L1 [52]	L2 [52]
logβ	8.8	18.0 [58]	14.8 [55]	14.7 [59]	18.8	14.7	15.22

## Data Availability

No new data were created or analyzed in this study. Data sharing is not applicable to this article.

## Abbreviations

DOTMP	1,4,7,10-tetraazacyclododecane-1,4,7,10-tetramethylenephosphonic acid
TCMC/DOTAM	2-[4,7,10-tris(2-amino-2-oxoethyl)-1,4,7,10-tetrazacyclododec-1-yl]acetamide
DOTP	1,4,7,10-Tetraazacyclododecane-1,4,7,10-tetra(methylene phosphonic) acid
BAPTA	1,2-bis(o-aminophenoxy)ethane-N,N,N′,N′-tetraacetic acid
cyclen	1,4,7,10-tetraazacyclododecane
DOTA	2,2′,2″,2‴-(1,4,7,10-Tetraazacyclododecane-1,4,7,10-tetrayl)tetraacetic acid
NOTA	2,2′,2″-(1,4,7-triazacyclononane-1,4,7-triyl)triacetic acid
HEHA	1,4,7,10,13,16-hexaazacyclooctadecane-N,N′,N″,N‴,N⁗,N″‴-hexaacetic acid
TETA	1,4,8,11-Tetraazacyclotetradecane-1,4,8,11-tetraacetic acid
EDTMP	{Ethane-1,2-diylbis[nitrilobis(methylene)]}tetrakis(phosphonic acid)
NETA	{4-[2-(bis-carboxy-methylamino)-5-(4-nitrophenyl)pentyl]-7-carbo-xymethyl-[1,4,7]triazanonan-1-yl} acetic acid
p-SCN-Bn-TCMC	S-2-(4-Isothiocyanatobenzyl)-1,4,7,10-tetraaza-1,4,7,10-tetra(2-carbamoylmethyl)cyclododecane
DO3AM	2,2′,2″-(1,4,7,10-tetraazacyclododecane-1,4,7-triyl)triacetamide
DOTA-Re(Arg11)CCMSH	DOTA-Re(Arg11)(Cys3,4,10,d-Phe7)-αMSH3-13
PEPA	1,4,7,10,13-pentaazacyclopentadecane-N,N′,N″,N‴,N⁗-pentaacetic acid
EDTA	Ethylenediaminetetraacetic acid
CHX-A″-DTPA	[(R)-2-Amino-3-(4-isothiocyanatophenyl)propyl]-trans-(S,S)-cyclohexane-1,2-diamine-pentaacetic acid
ACAC	Acetylacetone (Pentane-2,4-dione)
en	Ethylenediamine (Ethane-1,2-diamine)
C-NE3TA	4-carboxymethyl-7-[2-(carboxymethyl-amino)-3-(4-nitro-phenyl)-propyl]-[1,4,7]triazonan-1-yl-acetic acid
DTPA	diethylenetriaminepentaacetic acid
AZEP-DTPA	cis-{2,7-bis-[bis-carboxymethyl-amino)-methyl]-azepan-1-yl}-acetic acid
NPTA	[{4-carboxymethyl-7-[2-(carboxymethylamino)-ethyl]-perhydro-1,4,7-triazonin-1-yl}-acetic acid
NETA	{4-[2-(bis(carboxymethyl)amino)ethyl]-7- carboxymethyl [1,4,7]triazonon-1-yl}-acetic acid
PIP-DTPA	cis-2,6-bis[N,N-bis(carboxymethyl)aminomethyl]-1-piperidineacetic acid
p-SCN-Bz-DOTA-GA	2,2′,2″-(10-(1-carboxy-4-((4-isothiocyanatobenzyl)amino)-4-oxobutyl)-1,4,7,10-tetraazacyclododecane-1,4,7-triyl)triacetic acid
EEDTA	Oxybis(ethylenenitrilo)tetraacetic acid (1,7-diaza-4-oxaheptane-l,l,7,7-tetra-acetic acid)
EGTA	ethylene glycol-bis(β-aminoethyl ether)-N,N,N′,N′-tetraacetic acid (3,12-Bis(carboxymethyl)-6,9-dioxa-3,12-diazatetradecane-1,14-dioic acid)
L1	5-[2-(N-2-fluorenyl)ethylamino]-2,5,8-triaza [9]-2,6-pyridinophane
THP-cyclen	1,1′,1″,1‴-(1,4,7,10-Tetraazacyclododecane-1,4,7,10-tetrayl) tetrakis(propan-2-ol) acid
VCAM1	vascular cell adhesion molecule 1
LP	Lone electron pair
REE	Rare earth elements
mAb	Monoclonal antibody
I.P.	intraperitoneal
I.V.	intravenous
RIT	Radioimmunotherapy
